# High Rejection Sensitivity in Patients With Somatoform Pain Disorder

**DOI:** 10.3389/fpsyt.2021.602981

**Published:** 2021-03-29

**Authors:** Yeliz Nacak, Eva Morawa, Yesim Erim

**Affiliations:** ^1^Department of Psychosomatic Medicine and Psychotherapy, University Hospital of Erlangen, Friedrich-Alexander University Erlangen-Nürnberg (FAU), Erlangen, Germany; ^2^Department of Psychosomatics and Psychotherapy, Faculty of Medicine, University Hospital of Cologne, University of Cologne, Cologne, Germany

**Keywords:** attachment, chronic pain, childhood adversities, adverse life events, rejection sensitivity, somatoform (pain) disorder

## Abstract

**Objective:** Rejection sensitivity (RS) is often associated with mental disorders but as yet has not been investigated in patients with somatoform pain disorder (SPD). The aim of the study was to explore the degree of RS in patients with SPD compared to healthy controls. In addition, we examined factors associated with RS and the moderator effect of SPD.

**Methods:** A total of 65 patients with SPD (confirmed by Structured Clinical Interview, SCID-I) and 65 age- and gender-matched healthy controls participated. Rejection Sensitivity Questionnaire (RSQ), Patient Health Questionnaire (PHQ-9, PHQ-15), Relationship Scale (ReSQ), Essen Trauma Inventory (ETI) and the Childhood Trauma Questionnaire (CTQ) were applied. Multiple linear regression analysis was performed.

**Results:** The level of RS was significantly higher in patients with SPD compared to healthy controls (M = 10.30, SD = 5.64; *M* = 6.13, SD = 2.50; *p* < 0.001; *d* = 0.95). Higher levels of depressive symptoms (*p* < 0.001), childhood adversities (*p* < 0.001) and the insecure attachment style (*p* = 0.007) were related to higher levels of RS. No significant moderation effect was detected.

**Conclusions:** Patients with SPD are highly sensitive to social rejection. In addition, insecure attachment styles as well as depressive symptoms and childhood adversities are strongly associated with RS. Further studies are needed to figure out how RS is connected to SPD over lifetime.

## Highlights

- Patients with somatoform pain disorder are highly sensitive to social rejection in comparison with healthy controls. Depression, childhood adversities and insecure attachment are strongly associated with rejection sensitivity.

## Introduction

Somatoform pain disorder (SPD) is defined by the presence of pain that either persists in the absence of a physical condition or is not fully explained by a medical condition. Psychological factors are central in the onset, severity, exacerbation and maintenance of SPD, according to the Diagnostic and Statistical Manual of Mental Disorders (DSM-IV) (1). The recent edition (DSM-5) introduced somatic symptom disorder (SSD) as a single diagnostic entity that replaces SPD and other somatoform disorders and no longer differentiates between medically unexplained or explained symptoms. Diagnostic criteria of SSD also included intensive thoughts, emotions, and behaviors in relation to the illness (2). In a nationally representative survey of the German general population, a 12-month prevalence rate of 3.2% has been found for SPD (3). Current models assume somatoform pain results from a complex interaction between bio- and psychosocial factors (4, 5) but the associated etiology and maintenance are still poorly understood. Therefore, it is necessary to identify associated factors for better understanding and future interventions.

### Rejection Sensitivity (RS)

The feeling of belonging is a fundamental human need to form and maintain secure and stable relationships (6). Social pain interferes with this human basic need and describes hurtful experiences as a result of interpersonal or social distress such as lovesickness, partners breaking up, bullying, ostracism, loss of a loved one, isolation, loneliness or rejection (7). The anticipation of rejection and increased distress when experiencing rejection has been defined in the literature of social psychology as Rejection Sensitivity (RS). RS is a cognitive-affective processing disposition of anxious expectation, readily having a perception and intense reaction to cues of rejection (8). Persons with high levels of RS perceive potential signs of social rejection more readily and interpret ambiguous signs as negative.

Concerning the clinical picture, patients with SPD often report a lack of understanding and helplessness among related persons or medical doctors, which leads them to feel rejected. Sometimes, even medical support or failure in the medical treatment would be perceived by patients as a form of rejection. This shows that patients with SPD are highly sensitive and distressed in social situations and also anticipate that they will be rejected by others (9, 10). Current research findings suggest similar factors and mechanisms are involved in the onset and maintenance of physical and social pain. For instance, the “pain overlap theory” of Eisenberger and Lieberman (11) postulates that a greater sensitivity to one type of pain might accompany a greater sensitivity to the other type of pain. Numerous neuroimaging studies have shown that experimentally induced physical and social pain experiences in a laboratory task share the same neurobiological pathways (12–14). This indicates that if someone suffers from chronic pain over the span of years, it might be assumed that they will be sensitive to the other kind of pain and vice versa. These findings suggest a strong link between SPD and social pain.

### Etiology of Rejection Sensitivity

The onset of a high RS seems to come from early childhood. Early and repetitive experiences of social pain like emotional neglect or abuse by primary caregivers may influence the development of RS (8). Downey and Feldmann (8) postulated that a high RS is a result of persistent and maladaptive experiences in childhood. Rosenbach and Renneberg (15) postulated that experiences of rejection in childhood and youth are considered a significant factor for being highly sensitive to rejection in later life. One recent study postulates also family and parenting factors, such as family conflict or maternal harshness, as predictive for RS (16). High RS seems to be strongly associated with the lack of secure attachment. Based on the interaction with primary caregivers during infancy and childhood, children develop a stable and secure internal “working model” of the self and others, which helps to predict and understand the responses of others and to establish future relationships (17). A secure attachment ensures the ability to manage distress and regulation of emotions, which have a protective effect on mental health and being related less to RS. However, an insecure attachment contributes dysfunctional regulation of stress and emotion (18) and may promote vulnerability to RS. In accordance with this, individuals with an insecure attachment style are more likely to report persistent perceived social pain, especially fearful attached individuals (19). Another study showed that insecure (fearful) attached students have higher RS values than secure attached students (20). In sum, these results provide support to the finding that insecurely attached individuals are more prone to feelings of social pain.

### Associations Between Rejection Sensitivity and Mental Disorders

Severe or repeated experiences of social pain or rejection may elicit a negative view of the self and higher sensitivity in social situations, which is often associated with mental health problems. A study on patients with borderline personality disorder (BPD) reported higher levels of RS in comparison with healthy controls and postulated that RS is a core component in BPD (21). One recent review (22) also displays disturbances in RS across different personality disorders. Numerous studies have identified high levels of RS and hypervigilance for signs of rejection as predictors for depression (23–25). Results of a previous meta-analysis provide evidence for the association between RS and mental disorders, such as depression, anxiety, borderline personality disorder and eating and bodydysmorphic disorder (26). However, the association between RS and mental health problems seems to be bidirectional.

RS may elicit mental health problems and vice versa. Mental disorders also lead to being more sensitive in social situations. This may also provoke rejection sensitivity. In sum, current findings indicate that RS may play an important role in mental disorders. However, no study has explored the association between RS and somatoform pain. A few studies have already shown that patients with SPD have problems in the interpretation of social signs, deficits in Theory of Mind (ToM) and emotional awareness (27–29). Therefore, the focus of the present study was to investigate the role of RS in patients with SPD, compared to a healthy control group. Clarifying this hypothesis is important for the identification of potential targets for future treatment and prevention efforts. Based on previous findings, we aimed to investigate the association between insecure attachment and high RS. We also aimed to investigate whether socio-demographic and other factors are linked to RS. In addition, we also examined whether the presence of SPD moderates the relationship between depressive symptoms, traumatic experiences and RS.

The following hypotheses were tested:

- We hypothesized that patients with SPD display higher RS levels than healthy controls.
- We presumed that insecure attachment, depressive symptoms, childhood adversities, the presence of SPD, the number of experienced traumatic events and socio-demographic factors are associated with RS.
- We postulated a moderation effect of SPD: the presence of SPD would strengthen the relationship between depressive symptoms, attachment style, traumatic experiences and RS.
- In addition, we assumed that insecure-attached persons will have higher RS levels than secure-attached persons, irrespective of being patients or healthy controls.

## Methods

### Study Design and Procedure

Patients with pain disorders who have failed previous outpatient medical treatments and psychotherapy in primary care are admitted to the day clinic of Psychosomatic Medicine and/or to the Multidisciplinary Pain Center (MPC) of the Institute of Anesthesiology. In the abovementioned units from August 2014 to May 2015, we asked 100 patients with SPD to participate in the study. Of these, 65 patients agreed to participate and were available for analysis (response rate 65%). A further 35 declined participation because of several reasons (no time or interest, logistical reasons and burdening questions). Non-responders did not differ in gender proportion (21 female: 60%; *p* = 0.073) or age (*M* = 46.4; SD = 15.13, min 22y, max 65y; *p* = 0.065). We recruited 65 age- and gender-matched healthy controls through advertisements in the university library (info boards), and we also asked participants to invite additional people who could meet the participation criteria. For matching the age, we recruited a similar number of participants from the age classes, which we have defined before. General exclusion criteria for both groups were people younger than 18 years of age or older than 65 years. Further exclusion criteria were current alcohol or substance abuse, any major organic or psychotic disorder, people who had insufficient German language skills or those with any disabilities that impaired understanding of the study and questionnaires. All participants gave written informed consent. After the study inclusion, all participants were invited to take part in the Structured Clinical Interview for Axis I disorders (SCID-I for DSM-IV) (30), conducted by a trained psychologist. Inclusion criteria for the patient groups consisted of the diagnosis of “pain disorder associated with psychological factors” according to Code 307.80 of the Diagnostic and Statistical Manual of Mental Disorders (DSM-IV) (1), which is consistent with the International Classification of Diseases criteria (ICD-10) (31). For the control group, the initial screening questionnaire (with twelve items) of the SCID-I was performed. None of the controls met the criteria for a current or lifetime disorder. After the interview, all participants completed the questionnaires by themselves in a separate room (only the list of potentially traumatic life events was performed as an interview by the same psychologist, immediately after the SCID). The local ethics committee of the Friedrich-Alexander-University Erlangen-Nürnberg (FAU) approved the study (approval number: 46_14B). Non-overlapping findings of the obtained data have already been used for our previous publication (32).

### Measures

#### Rejection Sensitivity Questionnaire (RSQ)

The RSQ (8) was used to assess RS, which measures two components (expectations and anxiety) in 20 hypothetical situations, in which an individual is susceptible to rejection by an important other. The cognitive component of RS (rejection expectancy) is assessed by rating on a six-point Likert scale how strongly the person expects a response of either acceptance or rejection from others (1 = very unlikely; 6 = very likely). The affective component (rejection anxiety) is assessed on a 6-point Likert scale by how anxious or concerned the subject would be regarding this response (1 = very unconcerned; 6 = very concerned). Scoring total score was for each situation (item) by multiplying the level of rejection anxiety by the reverse of the level of rejection expectancy and then averaging the resultant values across the 20 scenarios. The mean scores for anxiety and expectation for rejection are ranged from 1 to 6, and the total score is ranged from 1 to 36. Higher scores reflect greater sensitivity to rejection. RSQ was adapted for application in clinical and non-clinical samples. In this study, we used an adapted 20-item German version of RSQ with well-documented internal consistency (α = 0.88) (33). Cronbach's alpha in the present study was 0.94 for the total score (for the subscales anxiety α = 0.93; expectations α = 0.92).

#### Relationship Scale Questionnaire (ReSQ)

The ReSQ (34) is a self-rating instrument to identify the adult attachment style. ReSQ consists of 30 items scored on a five-point Likert-scale and yields four attachment subscale scores: secure, preoccupied, fearful and dismissing attachment. The “secure” attachment indicated that the individuals have a positive view of themselves and others. The “preoccupied” attachment is defined by a negative view of the self and a positive view of others. The “fearful” attachment is defined by a negative view of self and others. The “dismissing” attachment style is described by a positive view of self and a negative view of others. The scores of subscales are obtained by calculating the mean score of the related items. For dichotomizing attachment style, we have summarized the subscales preoccupied, fearful and dismissing attachment as insecure attachment. The German version of ReSQ has well-documented psychometric properties (35). In our study, Cronbach's alpha was 79 for the total score.

#### Childhood Trauma Questionnaire (CTQ)

The CTQ (36) is a standardized self-report instrument that measures retrospectively the severity of five types of childhood adversities (emotional, sexual and physical abuse, emotional and physical neglect). We used the validated German version (37). Items are rated on a five-point Likert-scale with higher scores indicating more severe traumatic experiences. Subscale scores range from 5 to 25, and the total score ranged from 25 to 125. The German version of CTQ has good psychometric properties (38). In our study, the internal consistency for the total score was 77.

#### Essen Trauma Inventory (ETI)

The ETI (39) is a self-rating questionnaire that contains a list of 15 potentially traumatic events (personally experienced or witnessed) and items concerning the objective and subjective threat of life and symptoms on the subscales intrusion, hyperarousal, avoidance and dissociation. Clinically relevant symptomatology according to DSM-IV is indicated by the presence of one traumatic event, objective and subjective threat to life and also achieving a cut off ≥ 27 points for the total sum score of the subscales intrusion, hyperarousal and avoidance. In the present study, ETI was conducted as an interview to assess the number of potentially traumatic events. For ETI good psychometric properties have been demonstrated (40).

#### Patient Health Questionnaire (PHQ)

The severity of somatic symptoms was assessed using PHQ-15, a 15-item subscale of the German PHQ (41). The items include the most relevant DSM-IV somatic symptoms. The PHQ-15 score ranges from 1 to 30. The severity of depressive symptoms was evaluated with the subscale PHQ-9. Each of the nine items corresponds to one of the DSM-IV symptoms for major depressive disorder. The PHQ-9 score ranges from 0 to 27. Both PHQ-15 and PHQ-9 scores represent the severity level whereby a score of ≥5 is considered mild, ≥10 medium, and ≥15 severe. Psychometric properties of both subscales are well-documented (42, 43). For the present study, Cronbach's alpha for PHQ-15 was 0.88 and for PHQ-9.93.

#### Structured Clinical Interview (SCID-I)

SCID-I (30) is a semi-structured interview for detection of current and lifetime Axis-I diagnoses according to the DSM-IV criteria (1). The German version of Section G (somatoform disorders) was applied for validation of SPD. Due to the frequent comorbidity with depression, we also used Section D for mood disorders.

### Statistical Analysis

The necessary sample size was calculated with G^*^Power Analysis software program. A total of 64 patients would be required for 80% power, assuming a type one error rate of alpha 0.05 to detect differences with an effect size of *d* = 0.5 between patients and healthy controls. All analyses were conducted using SPSS v. 24.0 (SPSS, Inc., Chicago, USA). Prior to analyses, the data were checked for normal distribution using statistical and visual methods. The Shapiro-Wilk test was significant, but normal distribution can be assumed by the central limit theorem. Also, visual plots (regression of standardized residuals) showed no signs of violation of normality assumption. Missing items in PHQ-9 and PHQ-15 were replaced by the mean of the fulfilled items. Missing items in RSQ, ReSQ and CTQ were replaced by the mean of the fulfilled items respective to the subscale. Patients with more than 20% missing values in one of the self-report questionnaires were excluded from the related analysis. Data for descriptive analyses were shown as mean values, standard deviations, frequencies and percentage values. The Chi-square test was applied for categorical variables. For comparisons between the groups, we used the *t*-test for independent samples. For multiple comparisons (mean scores of RSQ), we used the Bonferroni correction for alpha adjustment (44). We also calculated Cohen's d for estimating the effect size. Multiple regression analysis (through using the enter method) was performed for the total sample. In the first step, we investigated the association between attachment style (dichotomized; secure = 0, insecure = 1), number of traumatic events, childhood adversities, depressive symptoms, presence of SPD (patient = 0; healthy controls = 1), sociodemographic characteristics age, gender (male = 0; female = 1), education (converted up to middle school into “low” = 0 and university entrance diploma into “high” = 1) and RS (criterion variable). To test whether the presence of SPD moderates the association between depressive symptoms, attachment style, childhood adversities and RS, we added three interaction terms (SPD^*^ PHQ-9; SPD^*^ ReSQ; SPD^*^ CTQ) to the regression model in a subsequent step. Prior to the regression analysis, appropriate assumptions were tested. Before testing the moderation effects, all metric variables were mean centered. Correlation analyses among the variables were calculated to test multicollinearity (by inspection of a pairwise correlation matrix). If correlation coefficients between two variables were higher than 0.70 (45), one of the variables was excluded from the regression model. In all analyses, a significance level of *p* < 0.05 was predetermined. For the patient group, a separate correlation analysis was calculated for pain duration (in years) and RS.

## Results

### Socio-Demographical and Clinical Characteristics

There were no significant differences between the two groups with respect to gender, age, partnership status, professional training, or nationality. Differences regarding education level and employment status were observed. Scores and statistical values are presented in Table 1.

**Table 1:** Comparison of demographic characteristics between patients and healthy controls.

	**Patients with somatoform pain disorder (*n* = 65)**	**Healthy controls (*n* = 65)**	**Statistics**
**Age (years)**			
Mean (SD)	47.5 (10.6)	43.9 (11.8)	n.s^a^
Range	18–65	20–65	
**Gender**			
Female	45 (69.2%)	49 (75.4%)	n.s^b^
Male	20 (30.8%)	16 (24.6%)	
**Partnership**			
Yes	50 (76.9%)	47 (72.3%)	n.s^b^
No	14 (21.5%)	18 (27.7%)	
Not reported	1 (1.5%)	–	
**Level of education**			
Without certificate	2 (3.1%)	–	0.003^**^^b^
Middle school	37 (58.5%)	25 (38.4%)	
University-entrance diplma	23 (35.4%)	37 (56.9%)	
Other	1 (1.5%)	2 (3.1%)	
Not reported	1 (1.5%)	1 (1.5%)	
**Professional training**			
Without graduate degree	5 (7.7%)	1 (1.5%)	n.s^b^
Apprenticeship	41 (63.1%)	36 (55.4%)	
University diploma	16 (24.6%)	26 (40.0%)	
Other	2 (3.1%)	2 (3.1%)	
Not reported	1 (1.5%)	–	
**Employment status**			
Currently employed	36 (55.4%)	43 (81.6%)	0.001^***^^b^
Retired	11 (16.9%)	1 (1.5%)	
On sick leave	15 (23.1%)	–	
Other	1 (1.5%)	9 (13.8%)	
Not reported	2 (3.1%)	–	
**Nationality**			
German	60 (92.3%)	63 (96.9%)	n.s^b^
Others	3 (4.6%)	2 (3.1%)	
Not reported	2 (3.1%)	–	

*** *p ≤ 0.001*;

** *p ≤ 0.01*;

a *t-test*;

b *Chi-square test*.

The mean pain duration in the patient group was 12.1 years in a range of 1–28 years (SD = 7.8). The healthy controls reported no acute or chronic pain. In the SCID-interview, all of the patients met the criteria of SPD. Furthermore, 87% of the patients with SPD presented a current depressive episode. Moreover, 55.5% were diagnosed with recurrent depressive disorder following the SCID-I interview.

### Rejection Sensitivity in the Study Groups

Compared to healthy controls, patients with SPD showed a higher RSQ total score (*M* = 10.30, SD = 5.64; *M* = 6.13, SD = 2.50; *p* < 0.001). Patients were significantly more anxious about rejection (*M* = 3.46, SD = 1.04; *M* = 2.86, SD = 0.083; *p* < 0.001) and rated the likelihood of being rejected higher than healthy controls (*M* = 2.84, SD = 0.80; *M* = 2.11, SD = 0.50; *p* < 0.001). Insecure attached individuals, regardless of patients or healthy controls, also showed also higher RSQ total score in comparison to secure attached individuals (*M* = 11.83, SD = 5.79; *M* = 6.26, SD = 2.67; *p* = 0.006). Insecure attached individuals also obtained significantly higher values in the subscales RSQ anxiety (*M* = 3.80, SD = 0.92; *M* = 2.82, *M* = 0.85) and RSQ expectation (*M* = 3.01, SD = 0.82; *M* = 2.19, SD = 0.58) when compared with secure-attached individuals. The effect size was medium to high. Mean scores of RS and statistical values are depicted in Table 2.

**Table 2:** Comparison of the mean scores of Rejection Sensitivity Questionnaire (RSQ).

	**Patients group (A)**	**Healthy controls (B)**	**Total sample of insecure attached (C)**	**Total sample of secure attached (D)**	**Statistics *p*-value; *t*-value; effect size**
**RSQ anxiety**					
M (SD)	3.46 (1.04)	2.86 (0.83)	3.80 (0.92)	2.82 (0.85)	A > B^***^; *t* = 3.54; *d* = 0.65 C>D^***^; t = −5.97; *d* = 1.11
**RSQ expectance**					
M (SD)	2.84 (0.80)	2.11 (0.50)	3.01 (0.82)	2.19 (0.54)	A>B^***^; *t* = 6.01; *d* = 1.11 C>D^***^; *t* = −6.61; *d* = 1.25
**RSQ total**					
M (SD)	10.30 (5.64)	6.13 (2.50)	11.83 (5.79)	6.27 (2.62)	A>B^***^; *t* = 5.16; *d* = 0.95 C>D^***^; *t* = −7.18; *d* = 1.35

*** *p ≤ 0.001 (after Bonferroni-adjustment; p ≤ 0.008); M, mean; SD, standard deviation; d, cohen's effect size; (A): n = 64; (B): n = 61; (C): n = 77; (D): n = 48*.

### Correlations Between Rejection Sensitivity and the Variables of Interest

Two variables showed a correlation coefficient > 0.70 (somatic and depressive symptoms) among each other. Based on statistical criteria (correlation coefficients higher than 0.70) (45) somatic symptoms (PHQ-15) were excluded from the analysis. We decided to exclude PHQ-15 from the analysis because somatic symptoms were already represented by the presence of SPD. In addition, including depressive symptoms (PHQ-9) in the analysis was relevant due to frequent comorbidity with SPD. High significant correlations between childhood adversities, the number of traumatic life events, depressive symptoms, and RS total score were observed. Pearsons correlation coefficients are presented in Table 3. No significant correlation between RS total score and pain duration for the patient group was observed.

**Table 3:** Correlation coefficients between Rejection Sensitivity Questionnaire and variables of interest.

**Variables**	**A**	**B**	**C**	**D**	**E**	**F**
**A** Rejection Sensitivity (RSQ)	1	0.608^***^	0.394^***^	0.677^***^	0.561^***^	0.053
**B** Childhood adversities (CTQ)		1	0.556^***^	0.612^***^	0.482^**^	0.282^**^
**C** Traumatic life events (ETI)			1	0.574^***^	0.508^***^	0.331^***^
**D** Depressive symptoms (PHQ-9)				1	0.767^***^	0.152
**E** Somatic symptoms (PHQ-15)					1	0.210^*^
**F** Age						1

* *p ≤ 0.05*;

** *p ≤ 0.01*;

*** *p ≤ 0.001 (two-tailed); Pearson correlations were conducted; RSQ, Rejection Sensitivity Questionnaire (total score); PHQ-9, Patients Health Questionnaire (depressive symptoms); PHQ-15, Patients Health Questionnaire (somatic symptoms); CTQ, Childhood Trauma Questionnaire; ETI, Essen Trauma Inventory (number of traumatic events), n = 112–128 (varying sample size)*.

### Multiple Regression Analysis and Moderation Effects

For exploring the association between RS and attachment, childhood adversities, number of traumatic experiences, depressive symptoms, presence of SPD and sociodemographic variables (gender, age and education) and the moderation effect of SPD, multiple linear regression analysis was performed for the total sample. The first model explained 63.2% (R^2^ adj = 0.602, *F* = 21.07; *p* < 0.001) of the variance regarding RS with depressive symptoms (ß = 0.547; *p* < 0.001), childhood adversities (ß = 0.314; *p* < 0.001) and (insecure) attachment style (ß = 0.217; *p* = 0.007) as significant predictors (Table 4). Higher levels of depressive symptomatology, more childhood adversities and insecure attachment style were associated with higher RS levels. Based on the second model with the presence of SPD as a moderation variable (R^2^ = 0.645; R^2^ adj = 0.604; *F* = 15.692; *p* < 0.001), SPD did not moderate the relationship between depression, attachment style, childhood adversities and RS. The main effects of depressive symptoms, insecure attachment and childhood adversities remained in the model and showed a significant contribution (Table 4).

**Table 4:** Multiple regression analysis predicting rejection sensitivity.

	**Model 1 with the main variables**						**Model 2 with the moderation effect of presence of SPD**					
	**R**^****2****^ **=** **0.632 (R**^****2****^**adj.=** **0.602)**						**R**^****2****^ **=** **0.645 (R**^****2****^**adj** **=** **0.604)**					
**Independent variables**	**B^a^**	**SE^b^**	**β**	**t**	**p**	**VIF^c^**	**B^a^**	**SE^b^**	**β**	**t**	**p**	**VIF^c^**
Constant	5.720	2.032		2.814	0.006^**^		5.363	2.504		2.142	0.035*	
Depressive symptoms (A)	**0.418**	**0.079**	**0.547**	**5.296**	0.001^***^	2.844	**0.405**	**0.082**	**0.530**	**4.947**	0.001^***^	3.072
Childhood adversities (B)	**0.095**	**0.026**	**0.314**	**3.708**	0.001^***^	1.913	**0.113**	**0.031**	**0.373**	**3.601**	0.001^***^	2.865
Attachment style (C)	**2.284**	**0.826**	**0.217**	**2.764**	0.007^**^	1.644	**2.742**	**0.994**	**0.261**	**2.758**	0.007^**^	2.391
Presence of SPD (D)	1.405	0.937	0.138	1.499	0.137	2.258	−0.032	1.915	−0.193	−0.017	0.987	9.467
Number of traumatic events (E)	−0.219	0.223	−0.085	−0.985	0.327	1.979	−0.242	0.226	−0.094	−1.071	0.287	2.045
Education	−1.202	0.682	−0.118	−1.763	0.081	1.194	−1.213	0.687	−0.119	−1.766	0.081	1.218
Age	−0.032	0.031	−0.069	−1.027	0.307	1.221	−0.029	0.031	−0.064	−0.945	0.347	1.230
Gender	−0.458	0.723	−0.040	−0.633	0.528	1.064	−0.654	0.735	−0.057	−0.890	0.375	1.102
Interaction term (D) x (A)							−0.311	0.358	−0.072	−0.868	0.388	10.173
Interaction term (D) x (B)							−0.040	0.050	−0.073	−0.789	0.432	2.301
Interaction term (D) x (C)							−1.295	1.747	−0.059	−0.741	0.461	1.670

a *Regression coefficient*;

b *Standard error*;

c *Variance inflation factor; (A) Patients Health Questionnaire (PHQ-9); (B) Childhood Adversities Questionnaire (CTQ, total score); (C) Relationship Scale Questionnaire (ReSQ, 0: secure, 1: insecure); (D) 0: patient; 1: controls; (E) Essen Trauma Inventory (ETI); education = 0: “low” (up to middle school); 1: “high” (university diploma); gender (0: male, 1: female); Durbin Watson Statistik: 2.068, significant predictors are marked in bold*;

*** *p < 0.001*;

** *p < 0.01; n = 107. Significant predictors are marked in bold*.

## Discussion

To our best knowledge, this is the first study that examined RS in the context of SPD, using a clinical sample in comparison to healthy controls.

### High Rejection Sensitivity in Patients With Somatoform Pain and Insecure Attached Individuals

We investigated the level of RS in patients with SPD in contrast to healthy controls. In concordance with our hypothesis and clinical picture of SPD, patients with SPD demonstrated higher scores for RS than healthy controls. They were more anxious about rejection and also rated the likelihood of being rejected higher than healthy controls. These results are in line with a previous meta-analytic study, which estimated an association between RS and other mental health problems (26). Our results also support the pain overlap theory (12–14) which postulates that physical and social pain have similar underlying factors. In this study, patients showed greater sensitivity to RS, which is in concordance with the clinical picture of SPD. Prior studies have already shown that patients with SPD have difficulties in interpretation of social signs or emotional awareness (27–29). One possible explanation for the high levels of RS in patients with SPD could be the history of negative childhood adversities, which was also shown as etiological factors for RS in past researche (8, 15, 16). We also compared RSQ scores between groups of secure- and insecure-attached individuals, regardless of the presence of SPD. In this comparison, insecure-attached individuals scored also higher in RS than securely attached. They were significantly more anxious about rejection and estimated the likelihood of being rejected as higher. This suggests that the attachment style is a broader underlying factor for RS, regardless of a mental disorder, which has been already shown in earlier studies [f.e. (46)]. Sato et al. (46) have demonstrated that individuals with high levels of RS are more likely to be insecurely (anxiety) attached. The lack of a secure attachment seems to impair not only mental health but also the sensitivity of interpretation of socially ambiguous situations. Insecure attachments seem like an overlapping factor for both SPD and RS.

### Significant Association Between Rejection Sensitivity, Depression, Attachment, and Childhood Adversities

We assumed that depressive and somatic symptoms, insecure attachment style, childhood adversities and a number of traumatic experiences would have a strong association with RS. Our findings are consistent with our expectations. More than 60% of the variance was explained by depressive symptoms, childhood adversities and insecure attachment. This is in line with previous research [f.e. (8, 16, 19)]. One possible explanation may be that persons with depressive symptoms, like negative attribution and thoughts, may be more anxious about social rejection and more sensitive to cues of rejection. Furthermore, we assume that some depressive symptoms like difficulties in social interactions and withdrawal can provoke rejection and negative responses from/by others. In turn, this may strengthen the higher sensitivity to social rejection.

Findings of a recent review postulate this reciprocal interplay between social rejection and mental health problems (47). However, the positive association between RS and depressive symptoms may be in part attributed to the overlap of the same underlying symptoms. Recent research demonstrates that high levels of RS are associated with depression (23–25). For instance, Chango et al. (25) found that high RS in mid-adolescence could predict depressive symptoms in later adolescence. Also, one prospective study postulates RS as a vulnerability factor for later depressive symptoms (48). In our study, insecure attachment was also significantly associated with RS, which is similar to previous findings [f.e. (19, 20)]. Consistent with the attachment theory (17), RS may be elicited by repeated negative interpersonal experiences from (early) childhood. Our results also show that childhood adversities are significantly associated with RS. This result correlates well-with previous findings (15, 16, 49). Brendgen emphasized parental rejection and aggression and also peer rejection as a significant factor for RS (49). Downey and Feldman suggested a strong relationship between childhood adversities like persistent familiar aggression and RS (8). However, childhood adversities seem to have a strong association with high RS, which also seems to be a crucial factor in the etiology of SPD (32). Contrary to our hypothesis, the presence of SPD did not moderate the association between depressive symptoms, attachment style, childhood adversity and RS. This finding could have several possible explanations. One explanation could be the insufficient power of the model or the strong explanatory power of the main effects. Further research is needed to give more clarity.

### Strengths and Limitations

As the first study of its kind, we explored the association between RS and SPD, and we recruited a control group of similar age and gender distribution, which allows us to better discriminate the specifics of the patient group. While the present findings are important, additional research is needed to provide more insight and confirm the findings in greater depth. Our results should be interpreted within the scope of a cross-sectional design, whereby no causal relations can be postulated. Prospective studies should give more clarification and causality. One limitation is that we used self-reporting and retrospective instruments for the assessment of our study variables. Furthermore, a self-reporting instrument with fictive social situations measured RS. An experimental way to examine RS could be by a validated social exclusion paradigm, e.g., Cyberball (50). Moreover, differences among the subtypes of attachment concerning RS have not been investigated in the present study due to the small sample size for subgroup analyses. It is unclear how the different insecure attachment styles may affect RS. This should be investigated in future research. Furthermore, as the sample size calculation was conducted with respect to two-sample *t*-tests, it should be noted that the sample size might be too small to identify all relevant effects in the regression analysis. Therefore, corresponding results can be considered as preliminary and should be investigated further in future research. Almost all of our patients with SPD have a current depressive disorder. Therefore, the interaction between depression, RS, and SPD needs further analysis. Our findings are based on a comparison with a healthy control group. Further research can benefit from a comparison sample with someone also suffering from a mental disorder, and this allows us to differentiate between features that are more specific for SPD. In addition, we employed SCID-I for validation of the SPD according to the criteria of DSM-IV. In the fifth edition of DSM (2) the somatic symptom disorder (SSD) replaced the diagnosis of the somatoform pain disorder. SPD as a single entity is no longer in use. Also, other possible Axis-I and II disorders were not investigated and cannot be ruled out. Finally, we examined a selected patient group with high chronicity and symptom load, which searched for treatment in our clinic. It may be also interesting to investigate patients in the primary care setting.

## Clinical Implications and Conclusion

The present study gives first insights into the association between RS and SPD. The results suggest that patients with SPD may have difficulties in social situations, more precisely in situations of perceived social rejection. These results indicate the importance of RS in daily clinical practice with this patient group. Therapeutic treatments may improve patient sensitivity to rejection cues or interpretation of ambiguous situations more carefully. Several studies have shown the benefits of social support in various situations (51–53). One review (12) also demonstrated that social support has been associated with lower levels of perceived physical pain. As it is known that dimensions of insecure attachment moderate the relationship between social support and (mental and physical) health (54, 55), the attachment patterns should be considered according to the therapeutic treatment. Furthermore, insecure-attached individuals are sensitive to social rejection, regardless of the presence of SPD. In addition, an insecure attachment style, depressive symptoms and childhood adversities seem to play an important role for RS. Prospective studies are needed to figure out how RS is connected to SPD over the lifetime. Improving our knowledge would foster better-adapted therapeutic techniques.

## Data Availability Statement

The raw data supporting the conclusions of this article will be made available by the authors, without undue reservation, to any qualified researcher. Requests to access the dataset should be directed to yeliz.nacak@uk-koeln.

## Ethics Statement

The studies involving human participants were reviewed and approved by Local Ethics Committee of the Friedrich-Alexander-University Erlangen-Nürnberg (FAU) (approval number: 46_14B). The patients/participants provided their written informed consent to participate in this study.

## Author's Note

The present work was performed by YN in fulfillment of the requirements of the Friedrich-Alexander-University Erlangen-Nürnberg (FAU) for obtaining the degree Dr. rer. biol. hum.

## Author Contributions

YN and YE conceived of the presented idea. YN developed the theory and performed the computations. EM and YE verified the analytical methods. YN wrote the first draft of the manuscript. YE supervised the findings of this work. All authors contributed to the article and approved the submitted version.

## Conflict of Interest

The authors declare that the research was conducted in the absence of any commercial or financial relationships that could be construed as a potential conflict of interest.

## Abbreviations

SPD: Somatoform Pain Disorder
SSD: Somatic Symptom Disorder
RS: Rejection Sensitivity
SCID-I: Structured Clinical Interview for axis-I disorder
DSM: Diagnostic and Statistical Manual of Mental Disorders
RSQ: Rejection Sensitivity Questionnaire
ReSQ: Relationship Scale Questionnaire
CTQ: Childhood Trauma Questionnaire
ETI: Essen Trauma Inventory
PHQ: Patient Health Questionnaire.
